# Effect of UV irradiation on *Echinaceae purpureae* interactions with free radicals examined by an X-band (9.3 GHz) EPR spectroscopy

**DOI:** 10.1007/s00044-014-1170-2

**Published:** 2014-07-22

**Authors:** Paweł Ramos, Barbara Pilawa

**Affiliations:** Department of Biophysics, School of Pharmacy with the Division of Laboratory Medicine, Medical University of Silesia in Katowice, Jedności 8, 41-200 Sosnowiec, Poland

**Keywords:** UV irradiation, *Echinaceae purpureae*, Antioxidant, Free radicals, EPR spectroscopy

## Abstract

The effect of UVA (315–400 nm) irradiation on *Echinaceae purpureae* interactions with free radicals was examined by the use of electron paramagnetic resonance (EPR) spectroscopy. The changes of antioxidant properties of *E. purpureae* with time of UV irradiation from 10 to 110 min (10 min steps) were determined. DPPH as the paramagnetic reference was used in this study. Changes of EPR signals of the reference after interactions with nonirradiated and UV-irradiated *E. purpureae* were detected. Interactions of the tested *E. purpureae* samples caused decrease of the EPR signal of DPPH as the result of its antioxidant properties. The decrease of the amplitude of EPR line of DPPH was lower for interactions with UV-irradiated *E. purpureae*. EPR examination confirmed antioxidant properties of *E. purpureae*. The weaker antioxidant properties of *E. purpureae* after UV irradiation were pointed out. *E. purpureae* should be storage in the dark. The tests bring to light usefulness of electron paramagnetic resonance with microwave frequency of 9.3 GHz (an X-band) in examination of storage conditions of pharmacological herbs.

## Introduction

The ability of the food and pharmacological substances to interactions with free radicals is their important property (Pawłowska-Góral *et al*., 2013; Rzepecka-Stojko *et al*., 2012). The results of therapy depend on quenching of free radicals in living organism. Free radicals are responsible for a lot of negative effects in organism, and their inactivation is needed. Free radicals have unpaired electrons, which cause major biochemical reactions and destroy the structures in cells. Free radicals are dangerous during the diabetes, polyneuropathy, arteriosclerosis, and cancer (Eaton *et al*., 1998; Pryor, 1976; Bartosz, 2006). The substances used in medicine should not contain free radicals, and they should be antioxidants. Pharmacological species as antioxidants react with free radicals, which loss their unpaired electrons and become diamagnetic. The activity of diamagnetic molecules is lower than paramagnetic free radicals, the risk of modification of chemical structures in tissues decreases, and their functions are not destroyed (Jaroszyk, 2008; Bartosz, 2006).

The examination of contents of free radicals in food (Pawłowska-Góral *et al*., 2013), drugs (Ramos *et al*., 2013), herbs (Kurzeja *et al*., 2013), biopolymers (Chodurek *et al*., 2012), cells (Pawłowska-Góral and Pilawa, 2011), and tissues (Eaton *et al*., 1998; Bartosz, 2006) by electron paramagnetic resonance (EPR) is known. EPR spectra were obtained for coffee (Nemtanu *et al*., 2005), tea (Wawer and Zawadzka, 2004), meat (Sin *et al*., 2005), dry fruits (Yordanov and Pachowa, 2006), and flour (Shimoyama *et al*., 2006). Free radicals may appear in drugs during sterilization processes, and such conditions accompanied by production of these paramagnetic dangerous molecules should be reject. The interacting factors killing the microorganisms during sterilization of drugs are radiation or high temperature (Skowrońska *et al*., 2012; Wilczyński *et al*., 2012). EPR studies showed that gamma irradiation (Wilczyński *et al*., 2012) or heating of drugs (Skowrońska *et al*., 2012; Kościelniak-Ziemniak and Pilawa, 2012) or herbs (Pawłowska-Góral *et al*., 2013; Kurzeja *et al*., 2013) produce free radicals. EPR spectroscopy was used to determine the optimal condition of radiative (Wilczyński *et al*., 2012) and thermal sterilization of drugs (Skowrońska *et al*., 2012; Kościelniak-Ziemniak and Pilawa, 2012). Thermal sterilization of herbs also forms free radicals in their molecular units (Pawłowska-Góral *et al*., 2013; Kurzeja *et al*., 2013). Free radicals (Chodurek *et al*., 2012) and biradicals (Najder-Kozdrowska *et al*., 2010) were found by EPR method in melanin biopolymers, model melanins, and their complexes with metal ions and drugs (Najder-Kozdrowska *et al*., 2010). Free radical concentration in melanins increased after adding diamagnetic metal ions, and it was lower after complex formation between melanin and paramagnetic metal ions (Najder-Kozdrowska *et al*., 2010). EPR spectra were measured for tumor cells (Pawłowska-Góral and Pilawa, 2011) and tissues (Eaton *et al*., 1998; Pryor, 1976; Bartosz, 2006). Laser irradiation of tumor cells with photosensitizer changed parameters of their EPR spectra, and the changes depended on type of cells (Pilawa *et al*., 2006). This information was obtained by comparative analysis of EPR spectra of free radicals in food, drugs, or biological samples (Pawłowska-Góral *et al*., 2013; Skowrońska *et al*., 2012; Pilawa *et al*., 2006).

EPR method is mainly used to study paramagnetic samples containing free radicals, but it is also possible to test antioxidant properties of diamagnetic samples by microwave absorption in this spectroscopy (Arshad *et al*., 2013; Rzepecka-Stojko *et al*., 2012; Eaton *et al*., 1998). The antioxidative interactions of the samples reflect the quench of EPR line of the paramagnetic reference after addition to its environment the tested molecules (Bartosz, 2006). For example, it is known as EPR measurement of antioxidative properties of bee pollen extracts (Rzepecka-Stojko *et al*., 2012) and *Morus* *Alba* Leaves (Kurzeja *et al*., 2013). The aim of this work was to show spectroscopic examination of the influence of UV irradiation on interactions of *Echinaceae purpureae* with free radicals. The effect of time irradiation on *E.* *purpureae*—free radicals interactions—was determined. The susceptibility of the antioxidative properties of tested drug on UV irradiation was checked to obtain practical knowledge about storage conditions for *E.* *purpureae*. The application of EPR spectroscopy to solve this problem was proposed.

## Experimental method

### The studied samples

*Echinaceae purpureae* is the most popular herbal immune adjuvant (Ghedira *et al*., 2008; Schapowal, 2013). *E.* *purpureae* preparations are consumed mainly in autumn and winter, when we need additional protection against bacteria and viruses. *E. purpureae* contains caffeic acid derivatives, flavonoids, polyacetylenes, polysaccharides, and small amounts of essential oil. Herb is particularly valued because of an immune. *E. purpureae* also exhibits properties such as anti-inflammatory, antibacterial, antiviral, antifungal, antioxidant, diuretic, cholagogue, and antispasmodic, and stimulates the synthesis of collagen and elastin (Kočevar *et al*., 2012; Schapowal, 2013).

Internal use of *E. purpureae* is as follows. The herb is used as a natural body tonic and shortens it the duration of colds. It has the prophylactic effect and helps in the treatment of respiratory infections, flu, and tonsillitis. It is also recommended by recurrent infections of the urinary tract and inflammation of the ascending cholangitis (Kočevar et al. 2012; Moraes *et al*., 2011).

External use of *E. purpureae* is as follows. The herb is useful in healing wounds, ulcers, burns, frostbite, and pressure ulcers. It supports the treatment of acne, psoriasis, eczema and herpes, as well as inflammatory vagina and vulva (Kočevar *et al*., 2012; Moraes *et al*., 2011; Ghedira *et al*., 2008; Schapowal, 2013).

The infusions used in the form of lotions relieve inflammation of the throat, mouth, and gums.

In cosmetology, the herb is used as a moisturizer, regenerating, antioxidant, soothing irritation, and inflammation of the skin (Kočevar *et al*., 2012; Schapowal, 2013).

In this work, nonirradiated and UVA irradiated samples of *E. purpureae* were examined. *E. purpureae* was exposed to UVA during different times. We used the following times of irradiation: 10, 20, 30, 40, 50, 60, 70, 80, 90, 100, and 110 min. The irradiation was performed by the use of Medison 250 lamp with four radiators with power of 20 W. The UVA wavelengths (*λ*) were in the range of 315–400 nm. The *E. purpureae* was irradiated from the lamp—sample distance of 30 cm.

### EPR measurements

EPR spectroscopy with microwaves of frequency of 9.3 GHz from an X-band was applied in the examination of *E. purpureae* interactions with free radicals. The paramagnetic reference—DPPH (2,2-diphenyl-1-picrylo-hydrazyl)—was used as the model source of free radicals. EPR spectra of free radicals of DPPH in 10 % ethyl alcohol solution were measured. These spectra were compared with EPR spectra of DPPH in ethyl solution after adding of the tested nonirradiated and UV-irradiated *E. purpureae* samples. The antioxidative properties of the tested samples cause the decrease of amplitude of EPR line of DPPH. The quenching of the EPR lines of DPPH after addition of *E. purpureae* to the solution was observed. The measurements were done for the samples placed in the thin-walled glass tubes with the external diameter of 1 mm. The empty tubes did not contain paramagnetic impurities, and the EPR signals were not observed for them.

EPR spectrometer with magnetic modulation of 100 kHz produced by RADIOPAN Firm (Poznań, Poland) was used in this experiment. Microwave frequency was measured by MCM101 recorder of EPRAD Firm (Poznań, Poland). EPR spectra of DPPH were numerically detected as the first derivatives by the The Rapid Scan Unit of JAGMAR Firm (Kraków, Poland) linked with the EPR spectrometer. The short time of acquisition of the individual EPR line was equal to 1 s. To avoid microwave saturation of the EPR lines, the spectra were detected with low microwave power of 2.2 mW, which corresponds to 15 dB of attenuation. The total microwave power produced by klystron of the EPR spectrometer was 70 mW.

**Fig. 1:**
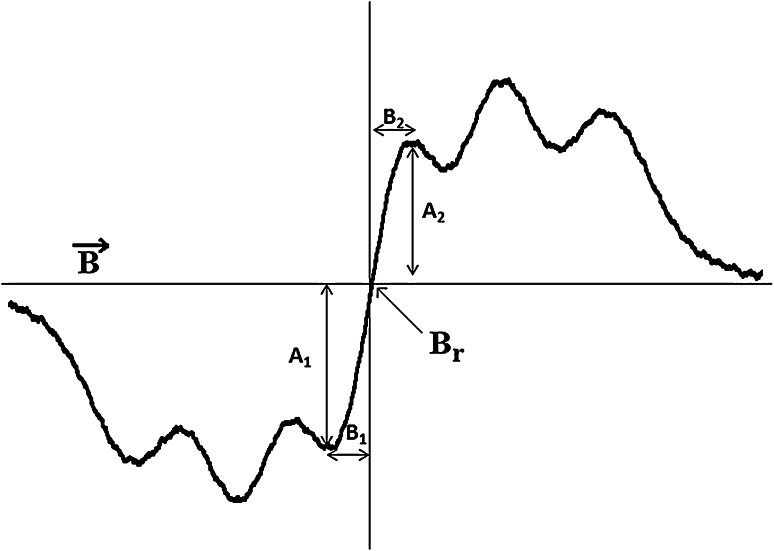
EPR spectrum of the reference—DPPH in ethyl alcohol solution. The parameters of *A*_1_, *A*_2_, *B*_1_, and *B*_2_ were used to analyze the asymmetry of EPR spectra. The asymmetry parameters—*A*_1_/*A*_2_, *A*_1_ − *A*_2_, *B*_1_/*B*_2_, and *B*_1_ − *B*_2_—were calculated.* B* is the magnetic induction of the field produced by electromagnet of the EPR spectrometer.* B*_*r*_ is the resonance magnetic induction

The EPR spectrum of the reference—DPPH in ethyl solution—is presented in Fig. 1. The analyzed lineshape parameters of this spectrum—*A*_1_, *A*_2_, *B*_1_, and *B*_2_—are shown in Fig. 1. Differences between *A*_1_ and *A*_2_, *B*_1_ and *B*_2_, indicate on asymmetry of the EPR spectrum. The values of *A*_1_/*A*_2_, *A*_1_ − *A*_2_, *B*_1_/*B*_2_, and *B*_1_ − *B*_2_, were calculated. Amplitudes (*A*) of the EPR spectra were obtained as *A*_1_ + *A*_2_. Amplitude of the EPR line increases with increasing of the free radical contents in the sample (Wertz and Bolton, 1986; Weil and Bolton, 2007). Linewidths (Δ*B*_pp_) of the EPR spectra were obtained as *B*_1_ + *B*_2_. Linewidths depend on magnetic interactions in the sample (Wertz and Bolton, 1986; Weil and Bolton, 2007). Dipolar interactions broaden EPR lines. In Fig. 1, the resonance magnetic field (*B*_r_) was marked. This value was used to obtain g-factor of free radicals existing in the source of free radicals—DPPH.

g-Factors were calculated from the paramagnetic resonance condition as (Wertz and Bolton, 1986) *g* = hν/μ_B_ *B*_r_, where h—Planck constant, ν—microwave frequency, μ_B_—Bohr magneton, and *B*_r_—induction of resonance magnetic field. g-Factor characterizes localization of unpaired electrons in the sample (Wertz and Bolton, 1986).

The professional programs were used to analyze the parameters of EPR spectra. The calculations were performed by the use of programs of JAGMAR Firm (Kraków, Poland) and LabVIEW 8.5 of National Instruments Firm.

## Results

**Fig. 2:**
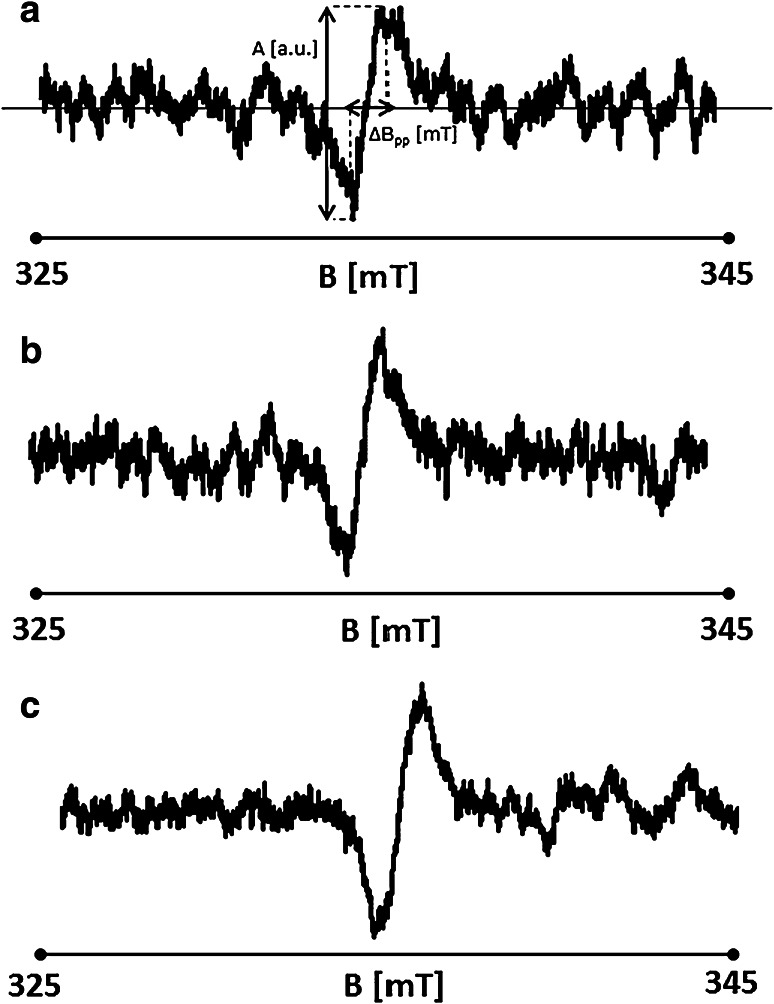
EPR spectra of DPPH in ethyl alcohol solution with *E. purpureae* nonirradiated (**a**), and UV irradiated during 10 (**b**), and 110 (**c**) minutes.* B* is the magnetic induction of the field produced by electromagnet of the EPR spectrometer

The comparison of the EPR spectra of DPPH in ethyl solution and DPPH in ethyl solution with *E. purpureae* indicates interactions between the tested herbs and free radicals. EPR spectrum of DPPH in ethyl solution with nonirradiated *E. purpureae* is shown in Fig. 2a. Amplitudes (*A*) and linewidth (Δ*B*_pp_) of EPR spectrum are marked. Amplitudes (*A*) and linewidth (Δ*B*_pp_) of DPPH line change upon interactions with *E. purpureae* (Figs. 1, 2). EPR spectra of DPPH in ethyl solution after adding of UV-irradiated *E. purpureae* for the herb exposed to electromagnetic waves during 10 and 110 min are presented in Fig. 2b, c, respectively. The shape and parameters of the EPR spectrum of DPPH changed after the addition of *E. purpureae* to the solution. The parameters of the EPR spectra of DPPH as the reference, and DPPH interacting with *E. purpureae* for the original—nonirradiated herb and the herb UV irradiated—are presented in Table 1.

**Table 1:** The analyzed parameters of the EPR spectra of the reference—DPPH interacting with nonirradiated and UV-irradiated *E. purpureae*

Sample	*A* [a.u.] (±0.1)	Δ*B*_pp_ [mT] (±0.02)	*A*_1_/*A*_2_ (±0.2)	*A*_1_ − *A*_2_ [a.u.] (±0.2)	*B*_1_/*B*_2_ (±0.02)	*B*_1_ − *B*_2_ [mT] (±0.04)
DPPH	10.4	0.49	1.1	0.5	1.24	0.05
Nonirradiated *Echinaceae purpureae*	0.8	0.48	1.2	0.1	0.62	−0.11
UV-irradiated *Echinaceae purpureae* during time (t):						
10 min	0.9	0.48	0.9	−0.1	0.90	−0.03
20 min	1.2	0.61	1.1	0.1	1.23	0.06
30 min	1.4	0.53	1.3	0.2	1.00	0.00
40 min	1.2	0.64	1.2	0.1	1.08	0.03
50 min	1.1	0.52	0.9	−0.1	1.36	0.08
60 min	1.1	0.54	1.1	0.1	0.61	−0.13
70 min	1.5	0.44	0.8	−0.1	0.86	−0.03
80 min	1.4	0.70	1.1	−0.1	0.64	−0.15
90 min	1.2	0.40	1.3	0.2	1.25	0.04
100 min	1.3	0.56	1.1	0.0	1.06	0.02
110 min	1.5	0.59	1.0	−0.1	0.86	−0.04

*A*—amplitude of the EPR spectra; Δ*B*pp—linewidth of the EPR spectra; lineshape parameters: *A*_1_/*A*_2_, *A*_1_ − *A*_2_, *B*_1_/*B*_2_, and *B*_1_ − *B*_2_. The parameters are defined in Fig. 1. The times (t) of UV irradiation of the sample are in the range of 10–110 min

g-Factors of 2.0036, typical for unpaired electrons localized on nitrogen atoms in DPPH, were obtained.

**Fig. 3:**
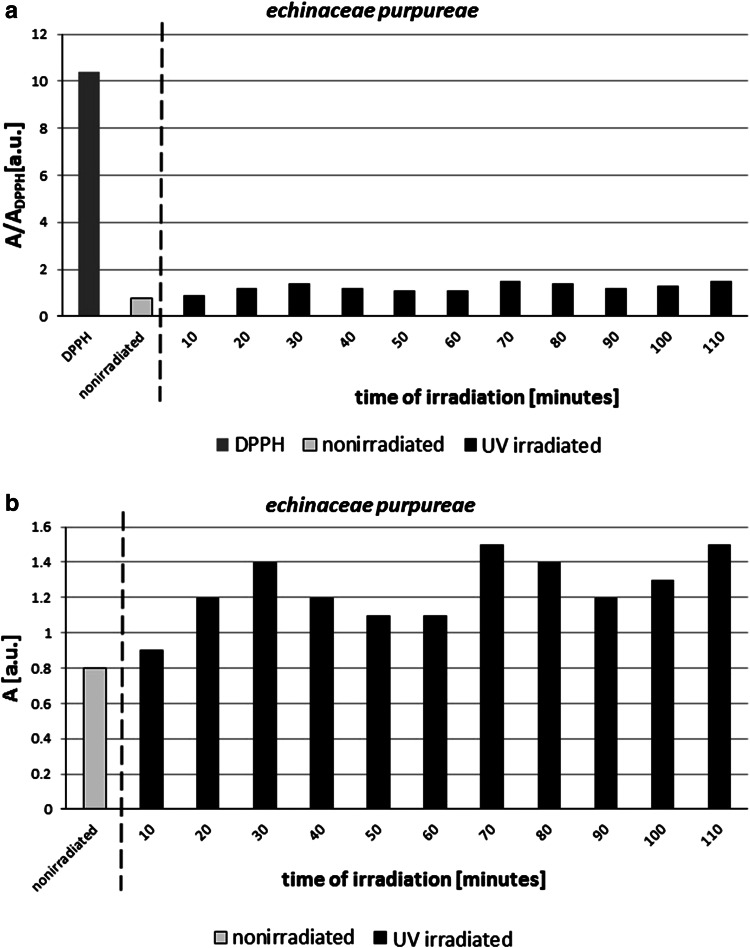
Amplitudes of EPR spectra of DPPH in ethyl alcohol solution, and DPPH interacting with nonirradiated and UV-irradiated *E. purpureae* in ethyl alcohol solution. The relative amplitudes A/ADPPH and the total amplitudes A are shown in Fig. 3a, b, respectively. A/ADPPH is the amplitude of EPR line of DPPH with the tested sample in alcohol solution divided by amplitude of EPR line of the reference—DPPH in ethyl alcohol solution. The total amplitude *A* is the amplitude of EPR line measured for DPPH in ethyl alcohol solution. The times (*t*) of UV irradiation of the sample are in the range of 10–110 min

The amplitude (*A*) of EPR lines of DPPH in ethyl alcohol solution with nonirradiated *E. purpureae* was lower than the amplitude of EPR signal of DPPH in ethyl alcohol solution, before adding of the tested herb (Table 1). Similar amplitude (*A*) characterizes UV-irradiated *E. purpureae* during time 10 min relative to the sample nonirradiated (Table 1). The higher amplitudes (*A*) of DPPH lines in ethyl alcohol solution were obtained for *E. purpureae* irradiated by UV longer than 10 min 20–110 min (Table 1). This correlation is presented in Fig. 3. From Fig. 3a, it is clearly visible that all the relative amplitudes (*A*/*A*_DPPH_) of EPR lines with the solution containing the tested herb are lower than one (Fig. 3a), so *E. purpureae* is antioxidant. UV irradiation negatively affects antioxidant properties of *E. purpureae* (Fig. 3a, b). In Fig. 3b, the total amplitudes (*A*) of DPPH interacting with nonirradiated and UV-irradiated *E. purpureae* are compared. The total amplitudes (*A*) are also lower for the UV-irradiated samples.

**Fig. 4:**
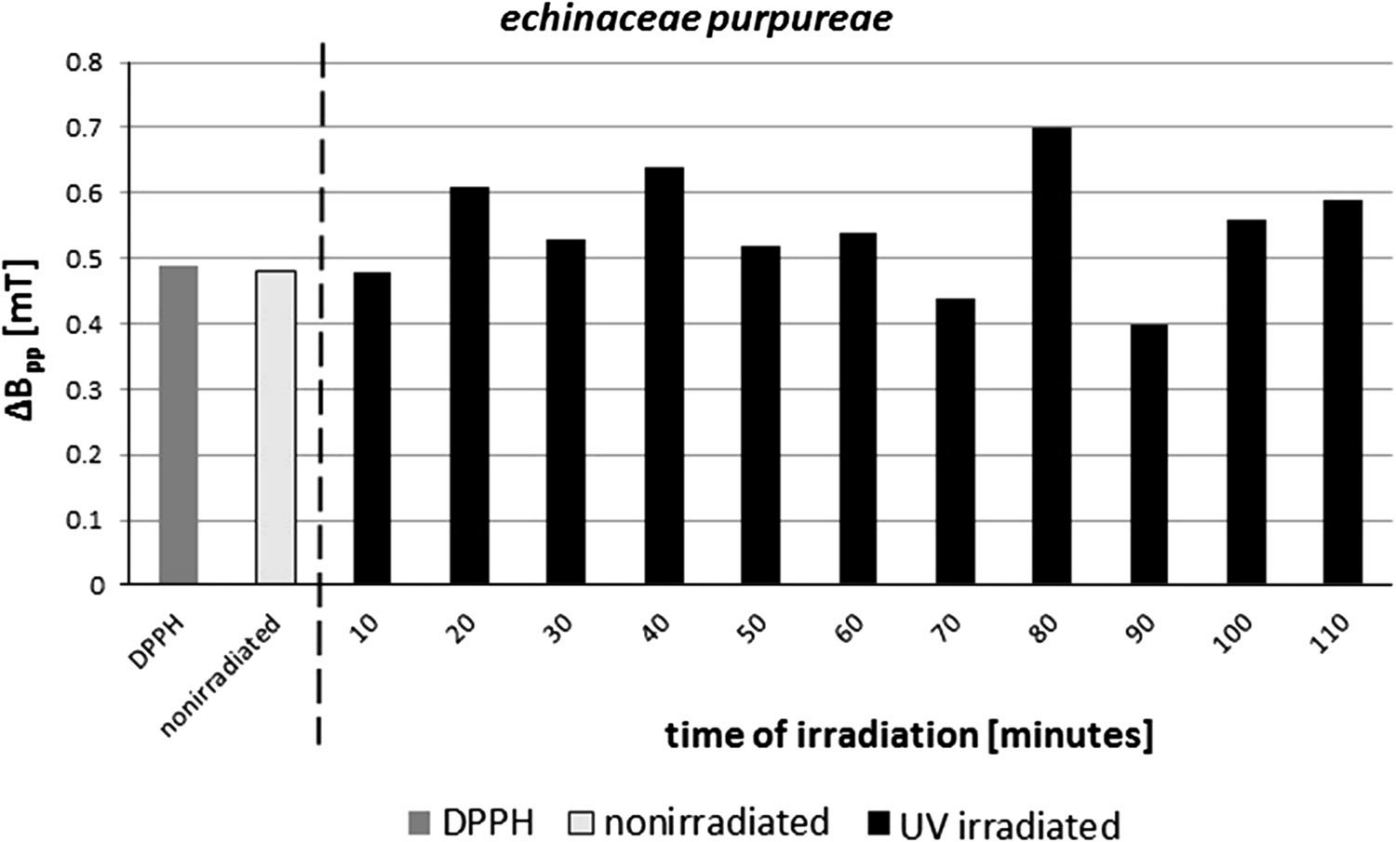
Linewidth (Δ*B*pp) of EPR spectra of DPPH in ethyl alcohol solution, and DPPH interacting with nonirradiated and UV-irradiated *E. purpureae* ethyl solution. A/ADPPH is the amplitude of EPR line of DPPH with the tested sample in alcohol solution divided by amplitude of EPR line of the reference—DPPH in ethyl alcohol solution. The total amplitude *A* is the amplitude of EPR line measured for DPPH in ethyl alcohol solution. The times (*t*) of UV irradiation of the sample are in the range of 10–110 min

The EPR spectra of DPPH in ethyl alcohol solution with *E. purpureae* were nonsymmetrical with the parameters of *A*_1_/*A*_2_ and *B*_1_/*B*_2_ which differ from 1, and the parameters of *A*_1_ − *A*_2_ and *B*_1_ − *B*_2_ differ from 0 (Table 1). It indicates that the major magnetic interactions exist in the tested samples. The parameters of lineshape of EPR spectrum of DPPH (*A*_1_/*A*_2_, *B*_1_/*B*_2_, *A*_1_ − *A*_2_, and *B*_1_ − *B*_2_) changed with the time of UV irradiation of *E. purpureae* (Table 1). The linewidths (Δ*B*_pp_) of EPR spectra of DPPH in ethyl alcohol solution both for nonirradiated and UV-irradiated *E. purpureae* had the high values (Table 1; Fig. 4). The linewidths (Δ*B*_pp_) changed with time of UV irradiation of the herbs. The changes were not regular, as shown clearly in Fig. 4. The complex magnetic interactions characterize the tested *E. purpureae.*

## Discussion

Application of EPR spectroscopy at the X-band (9.3 GHz) in food biophysics was confirmed. EPR spectra of the paramagnetic reference were used to determine antioxidative properties of the popular herb as *E. purpureae* (Kočevar *et al*., 2012; Moraes *et al*., 2011; Ghedira *et al*., 2008; Schapowal, 2013) with pharmacological interactions in human organism. The changes of shape and amplitudes of EPR spectra of DPPH in ethanol alcohol solution as the result of interactions of *E. purpureae* with free radicals of this reference were observed (Table 1; Figs. 2, 3, 4). The quenching of EPR lines of the reference by the tested herb (Fig. 3) brings to light its strong antioxidative interactions. The proposed method of examination of interactions of the herbs with free radicals has a lot of advantages. EPR spectroscopy is a physical method, which uses the EPR effect (Wertz and Bolton, 1986; Weil and Bolton, 2007). EPR effect is caused by Zeemann splitting of energy levels in magnetic field, and absorption of microwaves by electrons of the tested samples is studied. The energy of microwaves is fitted to the distances between the energy levels of electrons in magnetic fields. Electrons after absorption of electromagnetic waves with the respective frequencies are excited, and after they relax via spin–spin and spin–lattice relaxation processes (Wertz and Bolton, 1986; Weil and Bolton, 2007). In practice, the magnetic field is produced by electromagnet of the EPR spectrometer, and the tested samples are located in the resonance cavity. The absorption of microwaves is detected and numerically analyzed. The type of free radicals and concentrations may be determined (Wertz and Bolton, 1986; Weil and Bolton, 2007). The measurements needed only the low amount of the samples. Microwaves do not destroy the probes, and they may be tested several times. The EPR method is safe for the person who performs the studies. The economic costs of the EPR measurements at X-band are very low, because only the cold water is used to decrease the temperature of electromagnet that is needed and the electrical current. The parameters of the EPR spectra are analyzed numerically by the use of spectroscopic programs. Application of EPR in food biophysics (Pawłowska-Góral *et al*., 2013; Kurzeja *et al*., 2013), pharmacy (Skowrońska *et al*., 2012; Wilczyński *et al*., 2012), medicine (Pawłowska-Góral and Pilawa, 2011; Pilawa *et al*., 2006), biology (Pawłowska-Góral *et al*., 2013; Kurzeja *et al*., 2013), free radicals (Chodurek *et al*., 2012; Najder-Kozdrowska *et al*., 2010), techniques (Eaton *et al*., 1998; Wertz and Bolton, 1986), and biotechnology (Krztoń *et al*., 2009) is known. Our work is the fine example of usefulness of EPR spectroscopy in food biophysics.

The obtained results broaden our knowledge about antioxidative properties of the famous herb—*E. purpureae*. The effect of UV irradiation on interactions of *E. purpureae* was not physically studied so far, and our proposition of EPR analysis in this example has the innovatory character. The important result was obtained: the interactions of *E. purpureae* with free radicals decrease after UV irradiation (Table 1; Fig. 3), and this herb should not be stored in exposition to UVA. Only the short time of UV irradiation (10 min) does not negatively influence on antioxidative properties of *E. purpureae*, when the EPR lines of DPPH did not increase relatively to the nonirradiated herb (Table 1; Fig. 3). EPR parameters of DPPH changed with time of UV exposition (Table 1; Figs. 3, 4), so the antioxidative ability of *E. purpureae* evolutes in time. *E. purpureae* losts its antioxidative properties during UV exposition in time.

The interactions of *E. purpureae* with free radicals had a complex character, and this fact was reflected by the changes of linewidths (Δ*B*_pp_) (Fig. 4) and the asymmetry parameters (*A*_1_/*A*_2_, *B*_1_/*B*_2_, *A*_1_ − *A*_2_, and *B*_1_ − *B*_2_) of the DPPH spectra with time of UV irradiation (Table 1). These changes were not regular. The complex interactions are expected, because of the major transformations in *E. purpureae* under UV irradiation, when different chemical bonds may be broken and distances between unpaired electrons did not remain stable. The broadening of the EPR lines of DPPH interacting with *E. purpureae* is mainly caused by dipolar interactions between freer radicals.

The obtained results proved the possibilities of EPR studies of diamagnetic samples as *E. purpureae* by the use of paramagnetic probes—DPPH. The practical information about physical conditions of storage of *E. purpureae* was obtained. The economic aspects of EPR application in food biophysics were drawn.

## Conclusions

The performed studies of *E. purpureae* by the use of an X-band (9.3 GHz) EPR spectroscopy proved that

1. Nonirradiated and UV-irradiated *E. purpureae* reveal antioxidant properties; it interacts with free radicals and as the result, it causes decrease of EPR signal of the paramagnetic reference—DPPH in ethyl alcohol solution.
2. UV irradiation changes interactions of *E. purpureae* with free radicals, and it decreases the antioxidative properties of this herb.
3. The interactions of *E. purpureae* with free radicals depend on time of UV irradiation. The weaker interactions of *E. purpureae* with free radicals characterize the herb irradiated longer than 10 min (irradiated 20–110 min).
4. Taking to account of the antioxidative properties, *E. purpureae* should be stored without exposition on UV irradiations.
5. Usefulness of electron paramagnetic resonance spectroscopy with paramagnetic reference of DPPH to determine interactions of diamagnetic herbs with free radicals was confirmed.
